# A Novel Chinese Honey from *Amorpha fruticosa* L.: Nutritional Composition and Antioxidant Capacity In Vitro

**DOI:** 10.3390/molecules25215211

**Published:** 2020-11-09

**Authors:** Min Zhu, Haoan Zhao, Qian Wang, Fanhua Wu, Wei Cao

**Affiliations:** 1 School of Chemical Engineering, Northwest University, Xi’an 710069, China; minna9071@126.com (M.Z.); haoan_zhao@126.com (H.Z.); 18729197882@163.com (Q.W.); 2 College of Food Science and Technology, Northwest University, Xi’an 710069, China; wufanhua_1@163.com; 3 Bee Product Research Center of Shaanxi Province, Xi’an 710065, China

**Keywords:** physicochemical properties, antioxidant activities, nutritional composition, honey, polyphenol, *Amorpha fruticose* L.

## Abstract

False indigo (*Amorpha fruticosa* L., *A. fruticosa*) is the preferred tree indigenous for windbreak and sand control in Northwest China, while information on nutritional and bioactive characteristics of its honey is rare. Herein, 12 honey of *Amorpha fruticosa* L. (AFH) were sampled in Northwest China and the nutritional composition was determined. Sixteen mineral element and ten dominant polyphenols content were identified and quantified by ICP-MS (Inductively coupled plasma mass spectrometry) and HPLC-QTOF-MS (High performance liquid chromatography-Quadrupole time-of-flight mass spectrometry), respectively. Moreover, AFH demonstrated high levels of DPPH (1,1-Diphenyl-2-picrylhydrazyl) radical scavenging activity (IC50 100.41 ± 15.35 mg/mL), ferric reducing antioxidant power (2.04 ± 0.29 µmol FeSO_4_·7H_2_O/g), and ferrous ion-chelating activity (82.56 ± 16.01 mg Na_2_EDTA/kg), which were significantly associated with total phenolic contents (270.07 ± 27.15 mg GA/kg) and ascorbic acid contents (213.69 ± 27.87 mg/kg). The cell model verified that AFH exhibited dose-dependent preventive effects on pBR322 plasmid DNA and mouse lymphocyte DNA damage in response to oxidative stress. Taken together, our findings provide evidence for the future application of AFH as a potential antioxidant dietary in food industry.

## 1. Introduction

Honey is an important food that has long tradition of using as a natural sweetener, food preservative, and dietary supplement. Its main composition is a complex mixture of carbohydrates with proteins, enzymes, polyphenols, organic acid, free amino acids, minerals, and vitamins acting as minor components [1]. Variations of these components hinging on the floral and geographical origin of the honey and, as a result, honey from particular floral sources or regions has specific sensorial characteristics consisting of appearance, texture, aroma, and flavor with unique physicochemical properties (pH, electrical conductivity, acidity, moisture, sugar, protein contents, etc.) [2,3,4]. The research about the characteristic physicochemical parameters of monofloral honey has been studied for decades. Furthermore, various types of honey differ not only in their physicochemical properties but also in their different nutritious substances and values such as botanical origin-cased antioxidant ability changes. Researchers have demonstrated that phenolic compounds of honey have the capability of inhibiting or reducing the formation of free radicals, while providing antioxidant activity [5]. Polyphenol levels vary according to the honey botanical and geographical origin [6] in that the abilities of different types of honey against oxidation reactions are various while the nectar plants are numerous around the world [7,8,9]. Consequently, the characterizations of nutritious compounds and antioxidant effect in different types of honey are essential to enhance our information about the honey potential effects in human health.

China, owing its diverse conditions of geography and climate that provide a suitable environment for agricultural activity, has a long history of apiculture practices and diversiform honey resources. As a researching team focus on the bee product in China, we have operated many research studies concerning the monofloral honey in China, and revealed diverse characteristics on the physicochemical properties and therapeutic effect related to their botanical origin (Summarized in Table 1). However, the issues of physicochemical and bioactive properties of Chinese monofloral honey have, so far, been covered by limited scientific studies, commonly focused on the honey from original nectars such as acacia, jujube, buckwheat, chaste, etc. Throughout the whole country, there are still many varieties of nectar plants suitable for *Apis mellifera* bees to collect.

**Figure 1:**
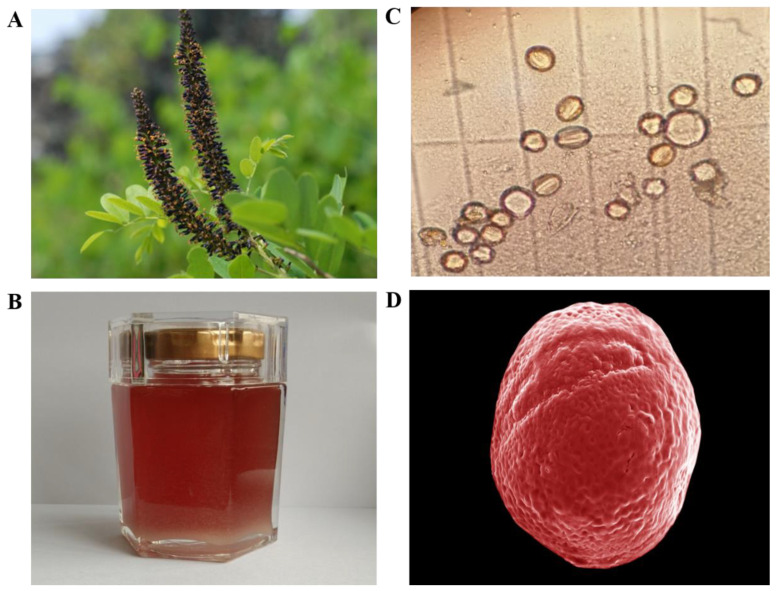
Flower, honey, and pollen of *Amorpha fruticosa* L. (**A**) Flower of *Amorpha fruticosa* L., (**B**) *Amorpha fruticosa* L. honey (AFH). (**C**) Microscope photomicrographs (×40) of pollen in AFH. (**D**) Scanning electron micrographs (SEM) of pollen in AFH. SEM experimental conditions: EHT: 5.00 KV. WD: 6.7 mm, Mag: 4.28KX, Signal A: Inlens.

During the investigation in Northwestern China, we found a particular nectar plant, named *Amorpha fruticosa* L. (*A. fruticosa*), characterised by the purple flowers in dense axillary as well as a terminal spike (Figure 1A). *A. fruticosa* belonged to the Leguminosae family was a perennial deciduous shrub and widely planted in China for erosion control and afforestation. Furthermore, research revealed that AF was rich in phytochemicals such as amorphin, which is a characteristic compound related to the antidiabetic and anticancer activities, suggesting the therapeutic potential of it against metabolic disease [21]. AF blooms May to August with scented flowers that are purplish blue with orange anthers and occur in upright spikes. The rich nectar production of these flowers with 10 protruding stamens with yellow anthers makes false indigo into a highly appreciated honey plant and important food source for bees [22,23]. As a result, honey from this plant is favoured for its amber appearance with a reddish tinge and special flavor (Figure 1B) as well as the potential folk medicinal purposes, such as gastrointestinal protection and wound healing [24]. However, to the best of our knowledge, the physicochemical and bioactive analysis for the characterization of the *Amorpha fruticosa* L. honey (AFH) type is still unknown.

Given the above, the present study aimed at typifying the AFH in China, according to the pollen spectrum and physicochemical parameters. Furthermore, phenolic profile and antioxidant activities will be determined to increase the current scarce knowledge about the bioactivities of AFH leading to further investigations with regard to developing the honey in health-promoting food applications.

## 2. Results and Discussion

### 2.1. Pollen Analysis

The results of microscopy pollen analysis were shown in Table 2. The data indicated that all the honey samples were monofloral since the proportion of *A. fruticosa* pollen in all samples varied from 58% to 73%. The micromorphology of the pollen from AFH was observed by microscope and SEM and was shown in Figure 1C,D, respectively, which were described as an ellipse (with a long axis of 14.96 µm and a short axis of 14.25 µm) with some grooves on the surface.

### 2.2. Physicochemical Analysis

**Moisture content.** Physicochemical parameters of samples are presented in Table 3. The moisture content is one of the key quality parameters of honey, as the high moisture content is an important factor concerned with the formation of a spoilage substance with an ensuing sour taste, named acetic acid [1]. The mean moisture content (expressed as a percentage of honey) in the investigated samples was 18.71%, which are in compliance with the imposed limit of ≤20% [25,26].

**pH.** The pH value of honey changes depending on the conditions during the extraction and storage period, resulting in the influences on texture, stability, and shelf life [1]. Although the limit has not been described in any regulations, honey pH should be low to avoid the microorganism contamination. The pH for all tested honey samples are acidic in character with an average of 3.96, which were acceptable and comparable with those obtained in other works [18].

**Free, lactonic, and total acidity.** Free acidity is an indicator of the deterioration of honey [1]. In terms of free acidity, the Codex Alimentarius Committee (2001) established a limit of 40 meq acid/kg for nectar honeys [25]. None of the studied samples has exceeded the maximum, while the mean value was 14.50 meq acid/kg. The mean value of lactonic acidity, as the acidity reserve, if the honeys become alkaline, was 3.57 meq acid/kg. Total acidity is the sum of free and lactonic acidities with a mean value of 18.07 meq acid/kg, indicating an absence of undesirable fermentation.

**Electrical conductivity.** The electrical conductivity of the honey is closely concerned with the contents of mineral salts, organic acids, and proteins. It is a reliable parameter for differentiating between honey types with different floral origins [1]. The results of studied honey samples showed mean electrical conductivity of 0.20 mS/cm. None of the samples exceeded the Codex maximum allowed of 0.8 mS/cm [25].

**Diastase activity and HMF (Hydroxymethylfurfural) content.** Diastase activity and HMF content were considered to be indicators of honey freshness because of their sensitivity to heating and storage factors [1]. The unprocessed honey samples exhibited low levels of HMF and high values of diastase activity with mean values of 0.32 mg/kg and 57.14 °Gothe, respectively. No samples exceeded the limits established by Codex Alimentarius Commission (2001) [25], the minimum value of 8 °Gothe for diastase activity and the maximum 40 mg/kg honey for HMF contents, suggesting the freshness of test samples.

**Color characteristics.** The Codex Alimentarius Committee (2001) stipulates that the color of honey should be nearly colorless to dark brown [25]. Several factors influence the honey color, including botanical origin, ash content, and storage conditions (temperature) [27]. The color of studied samples was analyzed by the values of L*, a*, and b. The L* value was used to estimate the degree of lightness, positive a* indicates red, negative a* present a green component, positive b* indicates yellow, and negative b* indicates a blue component. The mean L* values of the samples was 29.67, classifying AFH as the dark honey. All samples showed positive values of parameters a* and b*, indicating the red and yellow color presented in the honey types.

**Sugar content.** The concentration of fructose and glucose, which depends largely on the source of nectar, have been used as important indicators for the classification of monofloral honey [1]. As shown in Table 2, mean fructose and glucose values of AFH was 45.13% and 29.88%, respectively. The amount of glucose and fructose is higher than 60% for all samples in accordance with EC Directive 110/2001 [26]. Sucrose value is also an important parameter for honey quality, with the resulting Codex Alimentarius (2001) accepting the maximum sucrose content in honey as 5% [25]. AFH samples displayed a mean value of sucrose amount at 2.32%. No samples exceed the maximum limit of 5%.

**Protein content.** The mean value of protein content (mg/kg of honey) in AFH was 758.14 mg/kg, comparable to that found in Indian honey [28]. Proteins in honey are attributed to the presence of enzymes and free amino acids, which are introduced or derived from honeybees and floral sources. Thus, the protein of honey is variable.

**Proline content.** Proline represents a total of 50%–80% amino acids in honey, has been used as a parameter for the evaluation of the quality of honey, and an indication of adulteration when it falls below a value of 180 mg/kg [26], and, in some cases, adulteration with sugar [1]. All the samples studied had proline levels well above 180 mg/kg with the mean values of 318.17 mg/kg, indicating an absence of adulteration.

**Total phenolic contents (TPC)**. Polyphenols, which are an important group of compounds regarding the functional properties of honey, aroused high scientific and therapeutic interest recently [29]. TPC is considered to be a marker of honey’s antioxidant activity and generally used as an antioxidant test in many research studies. TPC of AFH from China are presented in Table 3. The mean value was observed at 270.07 mg GA/kg. The TPC of AFH samples was similar to that reported in Linden honey from Serbian (27.44 mg GA/100 g), Clover honey, and Rhododendron honey from Turkish (25.53 mg GA/100 g and 23.55 mg GA/100 g, respectively), but higher than that obtained in Homolje honey from Serbian (19.78 mg GA/100 g) and Acacia honey from Turkish (16.02 mg GA/100 g) [30,31].

**Ascorbic acid contents (ASAC)**. Aside from polyphenols, ascorbic acid also presents as an antioxidative substance in honey. It realizes antioxidant activity by interacting with a broad spectrum of ROS, suspending the chain reaction caused by free radicals as well as accelerating the regeneration of vitamin E [32,33]. Ascorbic acid content of AFH exhibited a mean value of 213.69 mg/kg (Table 3). Compared with other types of honey, AFH had a similar ascorbic acid content to that of Indian forest honey (260.90 mg/kg) and Algerian honey (236.80 mg/kg) [5,34], slightly higher than that of manuka honey (128.90 mg/kg), Malaysian pineapple honey (146.40 mg/kg), and Sundarban honey (117.04 mg/kg) [5,32,33], but significantly lower than that of *Robinia pseudoacacia* honey(843.70 mg/kg) and *Ziziphus jujuba* honey (1114.07 mg/kg) from China [35].

The physicochemical parameters of AFH were found to show approving accordance with honey quality regulations, indicating the good quality of AFH and that adulterations were not applied during the production processes.

### 2.3. Mineral Elements

Honey contains a variety of mineral elements, including macro elements (such as potassium, calcium, and sodium), trace minerals (such as iron, copper, zinc, and manganese), and heavy metals (such as lead). Major minerals are mainly derived from soil and nectar plants collected by bee, but consideration also needs to be given to environmental pollution, especially of aluminum, lead, and antimony, which may be unhealthful for humans and affect the quality and safety of honey [36]. Furthermore, as another nutritive component of honey, mineral elements perform a fundamental function in human biological systems by maintaining normal physiological responses, inducing the overall metabolism, influencing the circulatory system and reproduction, and as catalysts in various biochemical reactions [1]. Thus, the mineral element content can be considered as an important index of geographical origin and possible environmental pollution as well as a potential nutritive characteristic of honey [36].

The results of the mineral element determined in AFH samples are summarized in Table 4. Fifteen minerals were identified and quantified: potassium (K), calcium (Ca), sodium (Na), magnesium (Mg), zinc (Zn), iron (Fe), copper (Cu), nickel (Ni), chromium (Cr), cobalt (Co), molybdenum (Mo), aluminum (Al), lead (Pb), and antimony (Sb). Low concentrations of mineral content with the amount averaging 309.82 mg/kg found in our samples being responsible for the low values for electrical conductivity found in the current study (mean of 0.20 mS/cm) due to the positive correlation between them [37]. The K is the main mineral present in tested honey samples with a means of 250.024 mg/kg, and accounting for more than 75% of the total mineral quantified. Ca, Na, and Mg were present in moderate amounts in the samples with values varied from 19.438 mg/kg to 33.400 mg/kg, 18.072 mg/kg to 23.958 mg/kg, and 10.917 mg/kg to 12.727 mg/kg, respectively. The trace minerals of Zn, Fe, Mn, Cu, Ni, Cr, Co, and Mo were detected in all honey samples at low concentrations with the mean values of 0.683 mg/kg, 0.840 mg/kg, 0.110 mg/kg, 0.076 mg/kg, 0.035 mg/kg, 0.018 mg/kg, 0.003 mg/kg, and 0.009 mg/kg, respectively. The mineral profile of AFH was confirmed with that described by Hong et al. [38], who observed the *Amorpha fruticose* pollen collected by honeybees. Similarly, the predominant mineral of *Amorpha fruticose* bee pollen was K, followed by Ca, whereas Fe and Zn were present in a trace amount. The potential pollution minerals such as Al, Pb, and Sb were not detected in all AFH samples, which indicated consumption of AFH may not be associated with any public health problem caused by toxic elements. In comparison with other research studies, the contents found for K, Ca, Na, and Mg were similar to those of acacia honey from Vojvodina (Republic of Serbia) [39], but particularly less than those reported in many types of honey from Spain [27,36] and Morocco [3]. Although AFH could not be the main source of K, Ca, or Na for its low content of them, AFH could also be a good source for the trace element associated with bone health, immune function, adrenaline, and glucose metabolism, such as Ni and Cu, whose maximum recommended daily intake is 1 mg and 0.9 mg, respectively [40].

Furthermore, the macro elements obtained in the current study was significantly lower than that of jujube honey from the same geographical origin reported in our previous research [10], suggesting the low macro elements content could be a potential indicator to feature the AFH botanical origin. In the attempt to verify the surmise, furthers studies will be carried out that are constituted of comparisons with other botanical origin honey from the same geographical origin harvesting in the same year to avoid the influence of the soil, weather, and environment.

### 2.4. Characterization and Quantification of Phenolic Compounds in AFH

**Figure 2:**
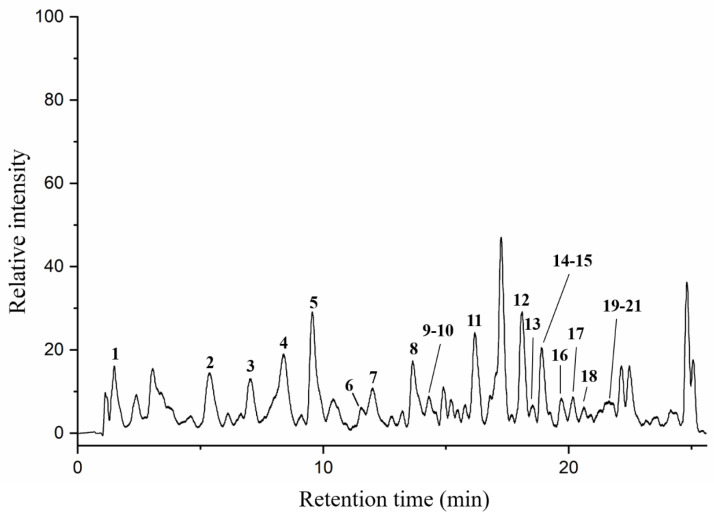
Total ion chromatogram (TIC) of AFH in a negative ion mode.

Particular phenolic acids and flavonoids, possessing antioxidant, anti-inflammatory, anti-cancer, and immune-enhancing capabilities, could make a significant contribution to health benefits of honey [41]. In this paper, twenty-one phenolic components in AFH were analyzed by HPLC-DAD/QTOF-MS along with their retention times (Tr), molecular formula, accurate comparisons between experimental ions, calculated ions (Calc), fragment ions, and references in negative ion modes. Ten of them were confirmed and quantified using available analytical standards. Two compounds: formononetin and chrysoeriol were discovered in nectar honey for the first time. Figure 2 demonstrated the total ion current (TIC) chromatogram of AFH, and the major peaks were assigned in Table 5. All compounds identified in AFH were mainly phenolic acids and flavonoids derivatives, and the process of identification and fragmentation was summarized as follows.

**Phenolic acids and derivatives.** Among the total number of nine phenolic acids and derivatives, the hydroxycinnamic acids, that is 4 caffeic acid, 6 cinnamic acid, 7 *p*-coumaric acid, 9 sinapic acid, and 10 ferulic acid, were predominant. Three of them, namely caffeic acid, *p*-coumaric acid, and ferulic acid, were identified based on the comparison of their retention times and characteristic MS spectral data with those of authentic standards. Cinnamic acid produced fragment ions at *m*/*z* = 129, *m*/*z* = 103, and *m*/*z* = 101, corresponding to the [M-H-H_2_O]^−^, [M-H-CO_2_]^−^ and [M-H-CO]^−^, respectively. Sinapic acid showed the fragment ions at *m*/*z* = 208 and *m*/*z* = 164, corresponding to the loss of CH_3_ molecule (15 Da) and CO_2_ molecule (44 Da) from the parent ion, respectively. Previous studies might provide evidence for these fragmentation behaviors [42,43]. In addition to the hydroxycinnamic acids, four hydroxybenzoic acids (including one gallic acid, two 4-hydroxybenzoic acids, three 2,4-dihydroxybenzoic acids, and five syringic acids) have been identified, which possessed the parent ions at *m*/*z* 169.0149, 137.0245, 153.0196, and 197.0455, respectively. In accordance with the literature [44], they exhibited the fragment ions at 125, 93, 109, 153, 119 and 179 due to losing carbon dioxide (*m*/*z* 44) or water molecules (*m*/*z* 18) from a negative ion mode because polyphenols contain one or more carboxyl or hydroxyl groups.

**Figure 3:**
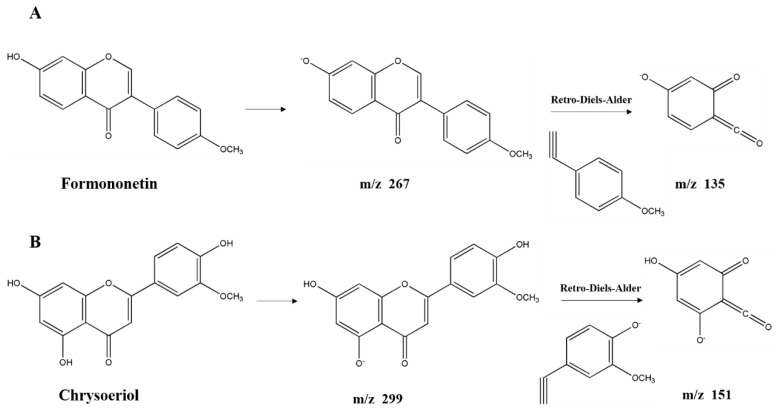
Structure of new compounds detected in AFH. Formononetin (**A**) and chrysoeriol (**B**).

**Flavonoids derivatives.** Twelve different polyphenols in the category of flavonoids derivatives, namely quercetin 3-*O*-glucosyl-rutinoside, rutin, quercetin, naringenin, apigenin 4′-*O*-glucoside, isorhamnetin, luteolin, diosmetin, formononetin, 3,3′,4′,5,5′,7-hexahydroxyflavanone, pinocembrin, and chrysoeriol (No. 8, 11, 12, 13, 14, 15, 16, 17, 18, 19, 20, 21) were detected in AFH. Five of them were confirmed and quantified using available standards. Quercetin 3-*O*-glucosyl-rutinoside and apigenin 4′-*O*-glucoside, the typical glycosylated flavonoids, produced fragment ions at *m*/*z* 609 and *m*/*z* 269, respectively, which generated by losing glucoside (*m*/*z* 162) [42]. Further loss of the rutinoside molecule from the precursor resulted in a fragment ion at *m*/*z* 301 of quercetin 3-*O*-glucosyl-rutinoside [44]. During the identification of flavonoids, especially in the absence of standards, MS data obtained from previous reports describing specific retro-Diels-Alder (RDA) fragmentation, were referred [45]. The fragment ion at *m*/*z* 151 was the characteristic fragment ion of some detected flavonoids (No. 12, 13, 15, 16, 17, 19, 20, 21) due to the RDA fragmentation pathway. Similar ion fragmentation information can be found in published literature [46], where the major fragments were generated from the loss of one or more water (*m*/*z* 18), carbon monoxide (*m*/*z* 28), and carbon dioxide (*m*/*z* 44). As the phenolic compounds first found in nectar honey, formononetin (Figure 3A) exhibited the parent ion at *m*/*z* 267.0666 and product ions at *m*/*z* 252, 223, 135, and 132. Chrysoeriol (Figure 3B), which is the flavonoid that has been identified in European honeydew honey [47], had the precursor ion [M − H]^−^ at *m*/*z* 299.0558 and fragment ions at *m*/*z* 284, 281, 271, 151, and 147. Given that the structure of formononetin is relatively similar to endogenous oestrogen (estradiol), formononetin is known to be one of the phytoestrogens, and gained the attention of researchers from the field of natural products, especially those working on anti-cancer drug discovery. Research studies have proved the elicits of antitumorigenic properties of formononetin via the tests in vitro and in vivo, suggesting the potential of it to be a promising candidate for prevention and therapy on cancers [48]. Moreover, formononetin was found as the main component implicated in the reported antimicrobial activity of red propolis due to its distinguished fungicidal and antibacterial activities [49]. Similarly, chrysoeriol is a flavonoid with three hydroxyl (OH) groups at 5, 7, and 4′ position, possessing distinguished antioxidant and lipase inhibitory activities [50,51]. On the other hand, formononetin and chrysoeriol were detected from the leaves of *A. fruticose* L. [23], and identified in nectar honey for the first time. All the results above suggested that these two compounds could be promising markers for botanical origin as well as the important compositions supporting the bioactivities of AFH. Compiling the results above highlights the potential of AFH to be a promising candidate for the chemoprevention and chemotherapy against the health-threat related to the oxidant stress, microorganisms, and cell apoptosis.

A total of 10 phenolic compounds were quantified based on extracted ion chromatograms (EIC) enabled different standards, and the contents as well as EIC are shown in Supplementary material in Table S1 and Figure S1. Syringic acid (36.43 ± 0.71 mg/kg) was the most abundant phenolic acid in the AFH, followed by ferulic acid, while rutin was the most abundant flavonoids with the content of 2.21 ± 0.01 mg/kg. AFH presented affluence in phenolic acid content, but scare in flavonoids. Compared with the edible fruit, AFH has a similar gallic acid and rutin content to that of banana and sea buckthorn berry, respectively, but significantly higher syringic acid than that of a sea buckthorn berry [52,53]. Compared with other types of honey, AFH had comparable quercetin content with acacia honey from Croatia [54], but lower quercetin content than *linen vine* honey from Cuba [55].

### 2.5. Antioxidant Analyses of AFH In Vitro

Antioxidant capacity is the ability and potential of honey to reduce oxidative reactions, which can cause deleterious changes within the food systems to produce adverse health effects [9]. Therefore, the antioxidant power of honey has been regarded as an eligible parameter that can couple with other measurements to evaluate the honey quality. Since the antioxidant activity cannot be evaluated by a single method, three different antioxidant tests (DPPH radical scavenging activity, ferric reducing antioxidant power (FRAP), and ferrous ion-chelating activity) were used to assess the antioxidant ability of AFH in our study. As shown in Table 3, the concentration of honey required to inhibit 50% of DPPH possessed a mean value of 100.41 mg/mL. Compared with other honeys studied in previous literatures, tested AFH samples showed a similar level of DPPH radical scavenging activity with that of Clover honey from Turkey (98.19 mg/mL) as the result of the similar TPC of them, but higher than Heather honey, Chaste honey, and Acacia honey from Turkey (123.56 mg/mL, 121.05 mg/mL, and 152.40 mg/mL, respectively) [31]. For the FRAP activity test, the mean value was 2.04 µmol FeSO_4_·7H_2_O/g, comparable to which of linen vine honey from Cuba and common eryngo honey from Turkey [29,31]. Ferrous ion-chelating activity is another index to evaluate the antioxidant properties due to the key role of Fe^2+^ playing in a Fenton reaction, which produces the hydroxyl radical and causes great oxidative stress damage to organisms [56]. In this paper, the ability of AFH to chelate ferrous ion was studied and expressed as Na_2_EDTA equivalents, of which mean value was 82.56 ± 16.01 mg Na_2_EDTA/kg honey. Our previous study has indicated that the flavonols with o-diphenolic groups in the 3,4-dihydroxy position in ring B and the ketol struc-ture, 4-oxo, 3-OH or 4-oxo, 5-OH in the C ring), such as rutin and quercetin, could chelate irons effectively and might exert their inhibitory effects [57]. As mentioned before, rutin and quercetin were major flavonoid compositions of AFH, and this may indicate that AFH possesses considerable chelating activity relating to the iron binding capacity of flavonoids.

**Figure 4:**
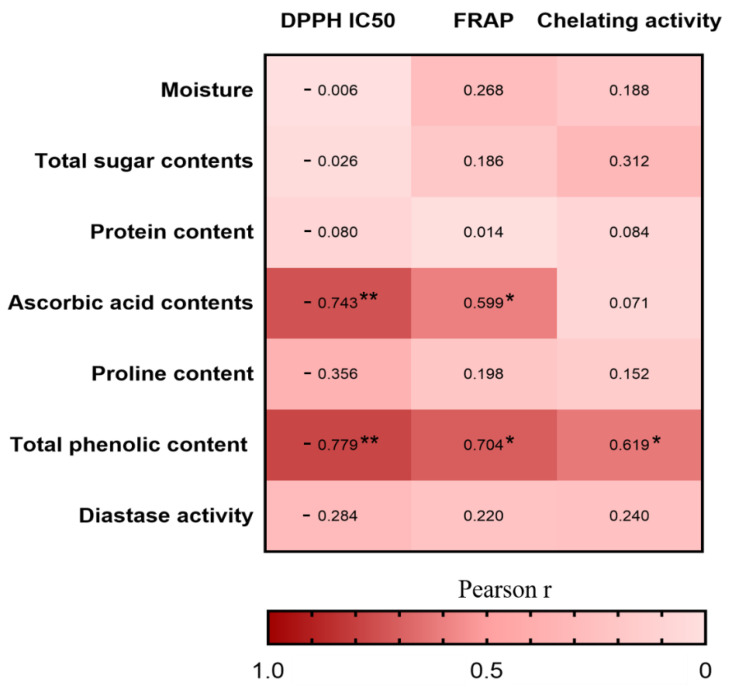
Heatmap analysis of the Pearson correlation between nutritional composition and antioxidant capacity in vitro. The values and intensity of the colors represent the degree of association between nutritional composition and antioxidant capacity in vitro. Statistical significance was measured by one-way ANOVA with the Tukey tests for multiple-group comparisons. * *p* < 0.05 and ** *p* < 0.01.

Furthermore, we investigated the Pearson correlations between the nutritional compositions and antioxidant activities in vitro (Figure 4). The correlation heatmap indicated that the TPC was positively associated with DPPH radical scavenging activity (r = −0.779, *p* < 0.01), FRAP (r = 0.704, *p* < 0.05), and chelating activity (r = 0.619, *p* < 0.05), whereas the ascorbic acid contents was positively associated with DPPH radical scavenging activity (r = −0.743, *p* < 0.01) and FRAP (r = 0.599, *p* < 0.05). The result is consistent with other studies in which the phenolic compounds and ascorbic acid are the main antioxidant substances in honey [58].

### 2.6. Assay for Effects of AFH on DNA Oxidative Damage

**Figure 5:**
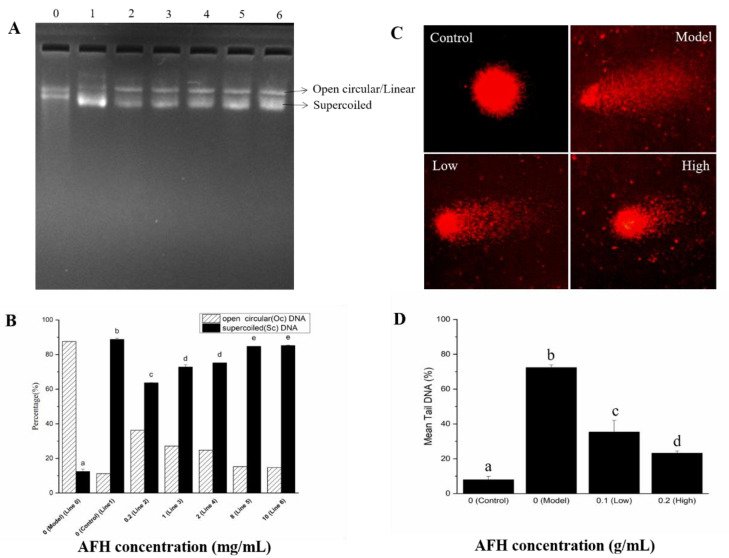
Protective effects of AFH on hydroxyl radical-mediated pBR 322 DNA strand breaks. (**A**): Gel electrophoresis assay. Line 0, 0.5 µg pBR 322 DNA + 1 µL of 1% H_2_O_2_ + 1 µL of 1.0 mM FeSO_4_ (DNA damage model group). Line1, only 0.5 µg pBR 322 DNA (normal DNA control group). Line 2–6, 0.5 µg pBR 322 DNA + 1 µL of 1% H_2_O_2_ + 1 µL of 1.0 mM FeSO_4_ + 4 µL 0.2 mg/mL, 1 mg/mL, 2 mg/mL, 8 mg/mL, and 10 mg/mL AFH, respectively. (**B**): Densitonetric analysis of supercoiled (SC) and open circular (OC) plasmid DNA of the gel electrophoresis. (**C**) Comet assay. Control: Normal mouse lymphocytes DNA, Model: Normal mouse lymphocytes DNA incubated with 200 mM H_2_O_2_ at 37 °C for 30 min. Low: Normal mouse lymphocytes DNA incubated with 0.1 g/mL AFH solution and 200 mM H_2_O_2_ at 37 °C for 30 min. High: Normal mouse lymphocytes DNA incubated with 0.2 g/mL AFH solution and 200 mM H_2_O_2_ at 37 °C for 30 min. (**D**) The mean proportion of tail DNA of the comet assay. Different lower case letters correspond to significant differences at *p* < 0.05.

**The protective effects of AFH on hydroxyl radical-mediated DNA strand breaks.** The protective effect of AFH on the DNA damage caused by oxidative stress was assessed using the pBR322 plasmid DNA breaks the system. Hydroxyl radical, generated from a Fenton reaction, can attack DNA molecules, resulting in the breaks of supercoiled plasmid DNA. The broken supercoiled plasmid DNA will appear in three forms including supercoiled (SC), open circular (OC), and linear (Linear). The effect of addition with a different concentration of AFH (0.2, 1, 2, 8, and 10 mg/mL) on the hydroxyl radical-induced DNA damage were shown in Figure 5A and Figure 5B, to determine whether the effects are concentration-dependent. The percentage of SC form in plasmid DNA decreased by 75.09% in the hydroxyl radical-induced line (Line 0) when compared with the control line (Line 1). The percentage of SC form was considerably reduced following the addition of AFH, revealing the effective protection of it on plasmid DNA from hydroxyl radical damage, and the effects showed a dose-dependent manner. Phenolic compositions may play a key role in the protective effect of honey on hydroxyl radical-induced plasmid DNA because of its scavenging hydroxyl radical activity and chelating ability, which can prevent the occurrence of the reaction and avoid DNA damage [15].

**Single-cell gel electrophoresis (SCGE) assay (Comet assay).** SCGE has gained in popularity as a standard laboratory technique for quantification of DNA oxidative damage. Breaks in the DNA caused by oxidation unfasten the supercoiling and allow DNA loops to expand, and move toward the anode on electrophoresis to form a path that resembles the shape of a comet with a brightly fluorescent head and tail. The length and intensity of the comet tail are proportional to the frequency of DNA breaks present in the cell [59]. As shown in Figure 5C,D, compared with the control group, pretreatment with hydrogen peroxide for 30 min of mouse lymphocytes significantly increased the proportion of tail DNA, suggesting evident damage of DNA. Hydrogen peroxide, which is a reactive product of oxygen metabolism, can react with ferrous ions in the cells and generates hydroxyl radicals to attack DNA [56]. However, a significant decrease in the proportion of mean tail DNA was found with increasing concentrations of AFH in a dose-dependent manner. Research studies have proven that phenolic compounds could chelate the ferrous ions and scavenge the hydroxyl radicals produced via the Fenton reaction [56]. Therefore, the scavenging effect and chelating ability of phenolic compounds in AFH was also surmised as a contribution to the prevention of DNA oxidative damage in mouse lymphocytes.

## 3. Materials and Methods

### 3.1. Honey Samples

**Figure 6:**
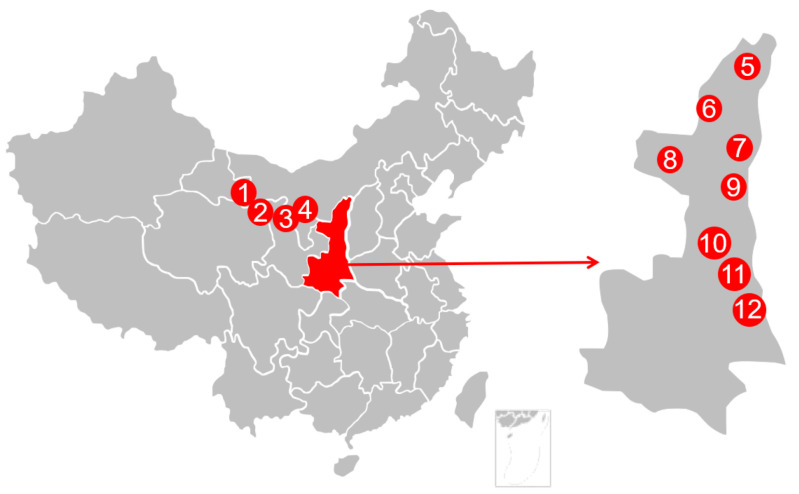
Geographical data for AFH’s sampling locations.

A total of twelve AFH samples were collected directly from honeycombs in Northwest China in 2018. All samples were transferred to the laboratory in their original packages and kept at 0 °C prior to analysis. The geographical information of samples was shown in Figure 6.

### 3.2. Pollen Analysis

The melissopalynological analysis was performed according to the method of Lutier and Vassiere [60], 5 g of diluted AFH was centrifuged at 10,000 rpm for 15 min, to separate the help of a brush on a slide containing a drop of lactophenol. Then, it was examined by a bright-field microscope (Olympus, Tokyo, Japan) at 400–1000 magnification. Scanning electron micrographs was observed by a Hitachi S-750 SEM system (Hitachi Company, Tokyo, Japan).

### 3.3. Physicochemical Analyses of AFH

All physicochemical parameters, including the glucose, fructose, and sucrose contents, were carried out according to the method established by the Association of Official Analytical Chemists (AOAC, 1990) [61]. The water content of honey was measured at ambient temperature using an Atago hand refractometer (Fuzhou Zhongtuo Optoelectronic Co., Ltd., China) and the readings were further corrected for a standard temperature of 20 °C by adding the correction factor of 0.00023/°C. The pH was measured with a pH meter (Delta 320, China) with a precision of ±0.01 pH units. Free lactonic and total acidity were determined by the titrimetric method and expressed as meq/kg. Electrical conductivity was measured at 20 °C in a Delta 326 conductometer. Diastase activity was determined using a UV751-GD Spectrophotometer. The diastase value was calculated using the time taken for the absorbance at 660 nm to reach 0.235 and was expressed in Gothe degrees. Hydroxymethylfurfural (HMF) content was measured based on a standard method using HPLC-UV (High performance liquid chromatography-Ultra violet), and the results were given in units of mg/kg. Sugar content was determined by HPLC with an RI (refractive index) detector (LC98-II R1, China). Proline was determined based on the reaction of proline with ninhydrin in an acidic medium, measuring the absorbance of resulting product at 517 nm.

Total phenolic content (TPC) was performed according to the Folin-Ciocalteu method described in our previous study [13]. A mixture of honey samples (1 mL, 0.1g/mL) with 1 mL of Folin-Ciocalteu reagent was placed under room temperature for 5 min. Then, sodium carbonate solution (5 mL, 1 M) and distilled water (3 mL) were added to the mixture. One hour later, the absorbance of 760 nm was measured. Gallic acid (GA) was used as a standard, and TPC is expressed as mg GA/kg honey.

The ascorbic acid content was estimated by the means described by Khalil and others [34]. Briefly, 100 mg of honey samples were extracted with metaphosphoric acid (1%, 10 mL) for 45 min and filtered at room temperature. Then, the filtrate (1 mL) was mixed with 2,6-dichlorophenolindophenol (DCPIP, 0.005%, 9 mL), and the absorbance at 515 nm was determined in 30 min. Ascorbic acid content was expressed as mg ascorbic acid/kg honey.

### 3.4. Mineral Elements Analysis

The mineral elements of AFH was estimated using inductively coupled plasma mass spectrometry (ICP-MS) based on the methods defined in Chinese National Food Safety Standards (GB 5009. 268–2016) [62]. Briefly, 0.5000 g of honey was dissolved in nitric acid (65%, 5 mL), and were allowed to digest under room temperature overnight. Then, the mixtures were dissolved in hydrogen peroxide (30%, 2 mL) before being digested at 140 °C for 2 h. The digestion was made up to 10 mL with nitric acid (2%) after being cooled. The solutions were centrifugated at 10,000 r/min for 10 min and the supernatant was kept to analysis.

Metal determination was determined using an inductively coupled plasma mass spectrometer (ICAPRQ, Thermo Fisher Scientific, MA, USA). The instrument was carried out with following conditions: RF power, 1500 W, plasma gas flow rate, 15 L/min, carrier gas flow rate, 0.8 L/min, auxiliary gas flow, 0.4 L/min, helium flow, 5.0 L/min, spray chamber temperature, 2 °C, and acquisition mode, spectrum. The content of each metal in honey was calculated with a corresponding standard curve, and expressed as mg/kg honey.

### 3.5. Characterization and Quantification of Phenolic Compounds in AFH

Each 20 g of the honey sample was thoroughly mixed with distilled water (pH = 2), and absorbed with a XAD-2 resin. The column was then washed with acidified water and distilled water at room temperature. Subsequently, the mixture was eluted with methanol, and evaporated under vacuum. Lastly, the residue was re-dissolved in HPLC-grade methanol of 4 mL. The methanol extracts were stored at 4 °C for further analysis after being filtered through an organic phase nylon filter.

The characterization of phenolic compounds in AFH was performed according to our previous literature [42], using Agilent 1200 Series Rapid Resolution HPLC system coupled with an Agilent Poroshell 120 EC-C18 column and Agilent 6510 ESI-Q-TOF-MS (Agilent Technologies, Satan Clara, CA, USA). The mobile phase was water (A) and methanol (B) using the following gradient elution: 0–2 min, 85% A, 2–10 min, 85–70% A, 10–25 min, 70–10% A, 25–30 min 10% A, 30–31 min, 10–85% A, and 31–45 min, 85% A. The injection volume was 2 µL, and the flow rate was set at 0.200 mL/min.

The tentative identification of phenolic compounds was performed based on the databases in published literatures in-house MS/MS database and HMDB Human Metabolome Database in terms of the comparisons between experimental ions, calculated ions (Calc), and fragment ions in the negative ion modes.

The quantification of major phenolic compounds in AFH were calculated based on the calibration curves of corresponding standards generated from the extracted ion chromatograms (EIC) of HPLC-DAD/QTOF-MS.

### 3.6. Antioxidant Activity In Vitro

#### 3.6.1. DPPH Radical Scavenging Activity

The DPPH radical scavenging activity was estimated using the method described in our previous study [13]. Honey samples were dissolved in methanol to obtain the honey solutions (0.1 g/mL). Different volumes of each honey solution were mixed with DPPH solution (0.1 mM, 4.0 mL), and the mixture was reacted darkly under room temperature for 30 min. The absorbances were measured using a spectrophotometer at 517 nm, and methanol was used as a blank. The percent inhibition of DPPH radical scavenging activity (RSA) of honey samples were calculated as follows.

RSA% = (A_c_ − A_s_)/A_c_ × 100

where A_c_ and A_s_ are the absorbencies of the blank and sample, respectively. The IC_50_ values was the concentration required of the honey sample when the inhibition reached 50%.

#### 3.6.2. Ferric Reducing Antioxidant Power (FRAP)

The ferric reducing antioxidant power was carried out by the means described in our previous study [13]. A solution of AFH (0.5 mL, 0.2mg/mL) was mixed with TPTZ (Tripyridyltriazine, 10 mM) and FeCl_3_ (20 mM) dissolved in acetate buffer (300 mM, pH 3.6). The absorbance of the mixture was determined at 593 nm. Trolox was used to prepare a standard curve and the FARP values of honey samples are expressed as mg Trolox/kg honey.

#### 3.6.3. Ferrous Ion-Chelating Activity

The ferrous ion-chelating activity of AFH was detected using the method reported by Cheng and others [56]. The reaction mixture, containing 0.2 mL of 0.1 g/mL honey sample solution, 0.1 mL of 1 mM FeSO_4_, and 0.3 mL of 1 mM ferrozine was adjusted to a total volume of 3 mL with methanol, mixed well, and allowed to stand at room temperature for 10 min. The absorbance of the mixture was measured at 562 nm against the blank. Na_2_EDTA was prepared for a standard curve and the result was expressed as mg Na_2_EDTA/kg honey.

#### 3.6.4. Assay for Effects of AFH on Hydroxyl Radical-Mediated DNA Strand Breaks

The protective effect of AFH on the extent of damage of DNA induced by a hydroxyl radical was investigated by the conversion of pBR322 DNA to open circular form based on the method described by Zhou [15] with some modifications. Briefly, 0.5 µg of DNA was treated with 1 µL of FeSO_4_ aqueous (1.0 mM), 1 µL hydrogen peroxide (1%), and 4 µL of an AFH sample at different concentrations (0.2, 1, 2, 8, and 10 mg/mL). Finally, the mixture volume was made up to 15 µL by phosphate buffer (50 mM, pH 7.0) and incubated in a water bath at 37 °C for 30 min. Then the mixture was loaded in an agarose gel (1%) and photographed by a gel imaging system. The result was quantified with the Quantity one program (version 4.6.2, BioRad Co, Hercules, CA, USA), while evaluation of antioxidant effects of AFH on DNA was done according to the increase or decrease of percentage of supercoiled DNA when compared with the control line. Experiments should be done in the dark to avoid the effects of photoexcitation of samples.

#### 3.6.5. Single-Cell Gel Electrophoresis (SCGE) Assay (Comet Assay)

Lymphocytes of healthy Kunming mouse were used and isolated as follows. The peripheral blood was mixed with lymphocyte separation medium (1:1, *v*/*v*) before centrifuging at 3500 rpm for 2 min. A pink layer at the middle of the separation medium was obtained, called lymphocytes. Mouse lymphocytes were suspended in phosphate buffer (0.15 M, pH 7.4) at a cell concentration of 1 × 10*/mL and viability exceeding 95%. Cell concentrations and viability were adjusted using Neubauer Improved Haemocytometer.

The SCGE assay were operated according to the method described in our previous literature [56] with a slight modification. In brief, lymphocytes were blended with hydrogen peroxide (200 mM) and different concentrations of AFH solution (dissolved in phosphate buffer), which was followed by incubation for 30 min at 37 °C in the dark. The mixtures were suspended in low melting agarose (0.7%) and applied to slides precoated with normal melting agarose (0.6%). Then, an additional layer of normal melting agarose (0.6%) was added upon the first two layers after the second layer of agarose was solid at 4 °C. After the agarose gel solidified, the slides were set in cell lysis solution for at least 1 h, which was followed by immersion and electrophoresis in electrophoresis buffer (300 mM, NaOH, 1 mM EDTA, pH 13) running at 25 V (300 mA) using a horizontal gel electrophoresis for 20 min in the dark. When electrophoresis finished, the slides were washed with neutralization buffer (0.4 M Tris/HCl, pH 7.5) to neutralize and then washed with ethanol for another 5 min to dehydrate. Ethidium bromide (30 mg/L, 20 µL) was dropped onto slides. The slides were covered with a cover slip and observed using a fluorescence microscope (Nikon 027012; Nikon, Tokyo, Japan). The result was scored and analyzed by an automated analysis system of the Comet Assay Software Project (CASP). The degree of DNA damage was evaluated based on the percentage of DNA in the tail, tail DNA % = (tail DNA/(head DNA + tail DNA)) × 100. At least 50 cells were scored from each slide and four slides were conducted per experimental group.

### 3.7. Statistical Analysis

Statistical analysis was carried out using SPSS version 24.0 (SPSS Inc., Chicago, IL, USA). Data analysis for comparisons among groups was determined by the means of the ANOVA procedure. Statistical analysis for significant differences among groups was estimated based on Duncan’s multiple range test. *p* < 0.05 was considered to be statistically significant. Correlations between the nutritional compositions and antioxidative activities in vitro were calculated by Pearson’s correlation test while the *p* values were corrected for multiple comparisons. Micromorphology of the pollen from AFH was processed by SEM photoshop.

## 4. Conclusions

The present study draws a detailed quality assessment picture of natural honey from *Amorpha fruticosa* L., which is a plant known for long-florescence and polyphenol-rich in China, in terms of physicochemical parameters (including moisture, pH, free acidity, lactonic acidity, total acidity, electrical conductivity, HMF, and diastase) and biochemical indexes (including sugar, protein, proline, mineral compositions, bioactive compounds, and antioxidant activity). Physicochemical and compositional analyses of AFH in the present work provide the first comprehensive data set to characterise this type of honey, suggesting that the qualities of AFH samples meet the requested criteria specified in international regulations, while phenolic compositions of formononetin and chrysoeriol, which are the flavonoids compounds found in *Amorpha fruticosa* L. leaves, could be the potential characteristics to feature the botanical origin of AFH samples. Moreover, our results revealed that AFH could be used as a potential health-promoting food for resisting oxidant damage and preventing pathogenesis of some diseases, due to its antioxidant activity and preventive effects on DNA oxidative damage.

## Figures and Tables

**Table 1:** List about different types of monofloral honey in China researched in the last 10 years. (Characteristic on physicochemical and biochemical properties as well as the therapeutic effect).

Types of Honey	Characteristics of Honey
Jujube (*Ziziphus jujuba* Mill.) Honey	Neutral pH (mean value of 6.71) [10], *Z. jujuba*-derived protein [11], Effect of induing apoptosis [12], Protective effects against chronic alcohol-induced liver damage [13].
Buckwheat (*Fagopyrum esculentum* Moench) Honey	High antioxidant capacity, Hepatoprotective effect [14], Protective effect of DNA [15].
Vitex (*Vitex negundo* Linna. Var. *heterophylla* Rehd) Honey	High caffeic acid content, Strong antioxidant activity, Hepatoprotection effect [16].
*Macleaya cordata* (Willd.) R. Br. Honey	Characteristic compositions of alkaloids [17].
*Prunella Vulgaris* Honey	The high content of rosmarinic acid, protective effects against colitis, modulative effect on gut microbial populations [18].
Other types (acacia; rape; chaste; etc.)	Amino acid could be an index to discriminate the botanical origin of jujube, rape, chaste acacia, and lungan honey [19], Chlorogenic acid and ellagic acid could be the major phenolic acid to identify the acacia honey adulterated with rape honey [20].

**Table 2:** Characterization of the analyzed AFH samples.

Samples	Botanical Source	Predominant Pollen (%)	Type of Honey	Production Year
1	*Amorpha fruticosa* L.	67 ± 5	Monofloral	2018
2	*Amorpha fruticosa* L.	58 ± 2	Monofloral	2018
3	*Amorpha fruticosa* L.	71 ± 4	Monofloral	2018
4	*Amorpha fruticosa* L.	59 ± 2	Monofloral	2018
5	*Amorpha fruticosa* L.	69 ± 9	Monofloral	2018
6	*Amorpha fruticosa* L.	64 ± 6	Monofloral	2018
7	*Amorpha fruticosa* L.	70 ± 5	Monofloral	2018
8	*Amorpha fruticosa* L.	65 ± 6	Monofloral	2018
9	*Amorpha fruticosa* L.	68 ± 4	Monofloral	2018
10	*Amorpha fruticosa* L.	62 ± 7	Monofloral	2018
11	*Amorpha fruticosa* L.	73 ± 4	Monofloral	2018
12	*Amorpha fruticosa* L.	69 ± 5	Monofloral	2018

Data expressed as means ± standard deviation.

**Table 3:** Results of physicochemical and bioactive analysis of honey samples.

Parameters	Values
Nutritional Composition	
Moisture (%)	18.71 ± 0.95
Fructose (%)	45.13 ± 3.13
Glucose (%)	29.88 ± 2.22
Sucrose (%)	2.32 ± 1.50
Total sugar (above three, %)	77.33 ± 4.66
Protein content (mg/kg)	758.14 ± 80.69
Ascorbic acid contents (mg/kg)	213.69 ± 27.87
Proline content (mg/kg)	318.17 ± 43.03
Total phenolic content (mg GA/kg)	270.07 ± 27.15
Diastase activity (°Gothe)	57.14 ± 7.80
Other Physiochemical Properties	
pH	3.96 ± 0.10
Electrical conductivity (mS/cm)	0.20 ± 0.00
L*	29.67 ± 2.92
a*	109.03 ± 11.26
b*	4.37 ± 2.18
Free acidity (meq/kg)	14.50 ± 0.61
Lactonic acidity (meq/kg)	3.57 ± 0.34
Total acidity (meq/kg)	18.07 ± 0.78
HMF (mg/kg)	0.32 ± 0.05
Antioxidant in vitro	
DPPH (IC50 mg/mL)	100.41 ± 15.35
FRAP (µmol FeSO_4_·7H_2_O/g)	2.04 ± 0.29
Chelating activity (mg Na_2_EDTA/kg)	82.56 ± 16.01

Data expressed as means ± standard deviation.

**Table 4:** Mineral contents of AFH (mg/kg).

	Content
K	250.024 ± 18.407
Ca	25.300 ± 4.360
Na	20.837 ± 2.043
Mg	11.886 ± 0.550
Zn	0.683 ± 0.518
Fe	0.840 ± 0.248
Mn	0.110 ± 0.021
Cu	0.076 ± 0.041
Ni	0.035 ± 0.003
Cr	0.018 ± 0.008
Co	0.003 ± 0.000
Mo	0.009 ± 0.001
Al	N.D.
Pb	N.D.
Sb	N.D.

Data expressed as means ± standard deviation, N.D.: not detected.

**Table 5:** Characterization of the phenolic components in AFH by HPLC-DAD/QTOF-MS.

Peak No.	Tentative Assignment	Tr (min)	[M − H]^−^ (*m*/*z*)	Molecular Formula	Calc *(m*/*z*)	Error (ppm)	Fragment Ions (*m*/*z*)
1	Gallic acid *	2.34	169.0149	C_7_H_6_O_5_	169.0142	4.24	169(100);125(75)
2	4-Hydroxybenzoic acid	5.48	137.0245	C_7_H_6_O_3_	137.0244	0.45	119 (25); 93 (25)
3	2,4-Dihydroxybenzoic acid	7.89	153.0196	C_7_H_6_O_4_	153.0193	1.96	153(100);109(20)
4	Caffeic acid *	8.76	179.0355	C_9_H_8_O_4_	179.0350	2.70	161(10); 135(50)
5	Syringic acid *	9.53	197.0457	C_9_H_10_O_5_	197.0455	0.99	179(40); 153(35)
6	Cinnamic acid	11.62	147.0451	C_9_H_8_O_2_	147.0452	−0.65	129(10);103(15)
7	*p*-Coumaric acid *	12.16	163.0404	C_9_H_8_O_3_	163.0401	2.26	119(30)
8	Quercetin 3-*O*-glucosyl-rutinoside	13.75	771.2022	C_33_H_40_O_21_	771.1989	4.28	609(15);301(5)
9	Sinapic acid	14.22	223.0621	C_9_H_10_O_4_	223.0612	4.13	208(10);179(10)
10	Ferulic acid *	14.28	193.0513	C_10_H_10_O_4_	193.0501	3.44	178(5);149(5)
11	Rutin *	15.74	609.1456	C_27_H_30_O_16_	609.1464	−0.82	301(12)
12	Quercetin *	18.31	301.0368	C_15_H_10_O_7_	301.0354	4.81	301(100);283(10); 273(10);151(5)
13	Naringenin *	18.52	271.0620	C_15_H_12_O_5_	271.0612	2.96	271(100);253(10); 243(30);227(10); 151(5); 119(5)
14	Apigenin 4′-*O*-glucoside	19.24	431.1010	C_21_H_20_O_10_	431.0984	3.49	431(100);268(10)
15	Isorhamnetin	19.65	315.0519	C_16_H_12_O_7_	315.0510	2.70	315(100);300(5); 297(10);271(10) 151(5)
16	Luteolin *	19.79	285.0409	C_15_H_10_O_6_	285.0405	1.71	267(15);241(10) 151(5)
17	Diosmetin	20.42	299.0562	C_16_H_12_O_6_	299.0556	0.22	281(15);271(10) 255(10);151(5)
18	Formononetin **	20.69	267.0666	C_16_H_12_O_4_	267.0658	1.33	252(5);223(5) 135(10);132(5)
19	3,3′,4′,5,5′,7-hexahydroxyflavanone	21.31	319.0464	C_15_H_12_O_8_	319.0454	1.36	319(100);301(5); 167(10); 151(10)
20	Pinocembrin *	21.47	255.0665	C_15_H_12_O_4_	255.0663	0.73	255(100);237(20) 227(20);221(20);151(5)
21	Chrysoeriol **	21.63	299.0558	C_16_H_12_O_6_	299.0561	−1.05	284(5);281(12) 271(12);151(5) 147(5)

* Confirmed using standards and quantification. ** Discovered for the first time in nectar honey.

## Supplementary Materials

The following are available online, Figure S1: Extracted ion chromatograms (EIC) of quantified polyphenols in AFH, Table S1: Contents of the 10 polyphenols in AFH.
