# Supplementary material for: A Novel Chinese Honey from Amorpha fruticosa L.: Nutritional Composition and Antioxidant Capacity In Vitro
Source: Molecules. 2020 Nov 9;25(21):5211. doi: 10.3390/molecules25215211 (PMC7664916; doi:10.3390/molecules25215211)
Supplement: Supplementary file 1 [file molecules-25-05211-s001.pdf]

Supplementary materials

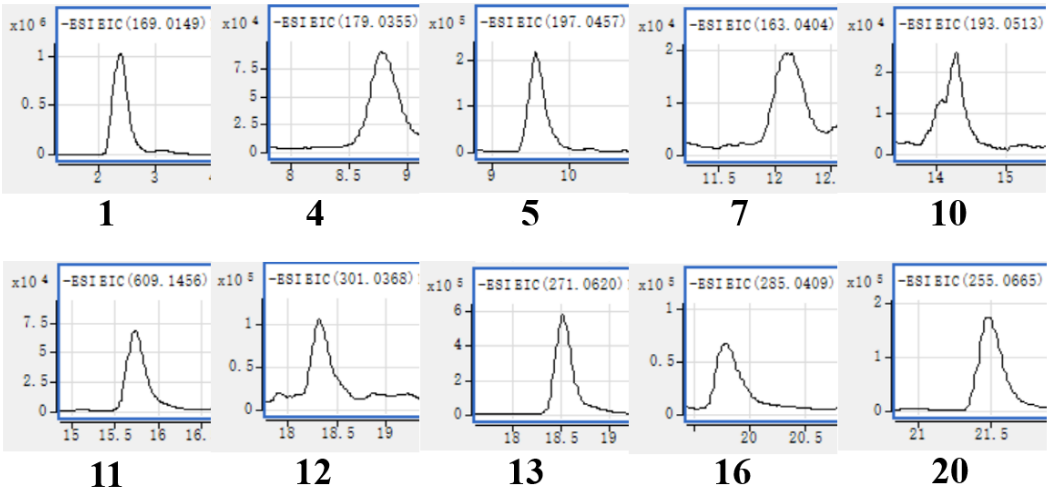

Supplementary Figure 1. Extracted ion chromatograms (EIC) of quantified polyphenols in AFH. (1) gallic acid; (4) caffeic acid; (5) syringic acid; (7) p-coumaric acid; (10) ferulic acid; (11) rutin; (12) quercetin; (13) naringenin; (16) luteolin; (20) pinocembrin.

Supplementary Table 1 Contents of the 10 polyphenols in AFH

Peak No.	Compounds	Contents (mg/kg)
1	Gallic acid	1.13±0.01
4	Caffeic acid	0.26±0.00
5	Syringic acid	36.43±0.71
7	*P*-Coumaric acid	1.46±0.01
10	Ferulic acid	1.89±0.13
11	Rutin	2.21±0.01
12	Quercetin	0.27±0.01
13	Naringenin	0.03±0.00
16	Luteolin	0.12±0.01
20	Pinocembrin	0.12±0.00

Peak No. corresponding to the number in Figure 2.
